# Anomalous electrical magnetochiral effect by chiral spin-cluster scattering

**DOI:** 10.1038/s41467-020-16751-2

**Published:** 2020-06-12

**Authors:** Hiroaki Ishizuka, Naoto Nagaosa

**Affiliations:** 1 Department of Applied Physics, The University of Tokyo, Bunkyo, Tokyo 113-8656 Japan; 2 RIKEN Center for Emergent Matter Sciences (CEMS), Wako, Saitama 351-0198 Japan

**Keywords:** Ferroelectrics and multiferroics, Spintronics

## Abstract

The non-collinear spin configurations give rise to many nontrivial phenomena related to the Berry phase. They are often related to the vector and scalar spin chiralities. The scalar spin chirality leads to the topological Hall effect in metals, while the vector spin chirality to the ferroelectricity of spin origin, i.e., multiferroics in insulators. However, the role of the vector spin chirality in conducting systems has not yet been elucidated. Here we show theoretically that the spin correlation with vector spin chirality in chiral magnets scatters electrons asymmetrically, resulting in nonreciprocal transport phenomena, i.e., electrical magnetochiral effect (eMCE). This asymmetric scattering appears in the leading-order scattering term, implying a large nonreciprocity in the charge and spin currents. We find that the temperature and magnetic field dependence of the eMCE reproduces that observed in MnSi. Our results reveal the microscopic mechanism of eMCE and its potential in producing a large nonreciprocal response.

## Abstract (web summary)

The microscopic mechanism for nonreciprocity of electron transport in bulk materials remains less well understood. Here, the authors show that asymmetric electron scattering by two-spin clusters induce nonreciprocal current (electric current proportional to the square of the electric field) in magnetic metals.

## Introduction

Non-coplanar magnetic configurations bring about rich electronic and magnetic properties of materials such as anomalous Hall effect^1–4^, orbital magnetization^5^, and electric polarization in insulators^6,7^. These studies revealed that the vector ***χ***_*ij*_ = **S**_*i*_ × **S**_*j*_ and scalar *χ*_*ijk*_ = (**S**_*i*_ × **S**_*j*_) ⋅ **S**_*k*_ spin chiralities are central concepts in the physics of non-collinear spin structures. Since the spin operator is odd in ${\mathcal{T}}$, *χ*_*ij*_ is even while *χ*_*ijk*_ is odd. Therefore, *χ*_*ijk*_ is related to the magneto-transport; topological Hall effect associated with *χ*_*ijk*_, both intrinsic^1,2^ and extrinsic^8–10^ mechanisms, are studied^11^. On the other hand, the inversion symmetry operation *P* about the center of the bond connecting *i* and *j* reverses the sign of ***χ***_*ij*_. The symmetry property implies ***χ***_*ij*_ is related to the electric polarization of spin origin in insulators^6,12^. In conducting systems, on the other hand, the broken *P* is subtle since the electric field in the metal is prohibited. However, there are several interesting nonreciprocal transport phenomena in noncentrosymmetric crystals^13–15^.

Nonreciprocal transport phenomena is a asymmetric dc electron transport, i.e., the current induced by a positive voltage is different from that by corresponding negative voltage. The reciprocal theorem by Onsager provides a basis to discuss the nonreciprocal linear responses^16,17^. This theorem originates from the time-reversal symmetry ${\mathcal{T}}$ of the microscopic dynamics, which is different from the macroscopic irreversibility. In transport theory, the Hermite symmetry also gives the reciprocal relation^18^, in addition to the space group symmetry of the crystal. Therefore, the breaking of *P* alone does not necessarily lead to nonreciprocal responses. The nonreciprocity becomes even more subtle and rich for the nonlinear responses^13,14^. The nonreciprocal dc transport in solids manifests in the *I*^2^ term of the *I*–*V* curve, where *I* is the injected electric current and *V* is the voltage drop^13,14^. For example, the *I*–*V* curve of eMCE follows^13^

1 $$V={R}_{0}(1+\gamma (B)IB)I,$$

which means that the external magnetic field is needed to break ${\mathcal{T}}$ for this effect. Recent experiments found the nonreciprocal response in various nonmagnetic materials such as Bi helix^13^, semiconductors subject to gate potential^14^, molecular conductors^19^, polar semiconductor^20^, and superconductor^21^. The nonreciprocal response also appears in magnetic materials, such as eMCE in metal/ferromagnet bilayer^22^, magnetic topological insulator^23^, and chiral magnets^24,25^. In the magnetic systems, the magnetic ordering and fluctuation seem to play a crucial role in sharp contrast to the band structure effects dominating in the nonmagnetic systems. Among them, a recent paper reported a detailed experiment on the temperature and magnetic field dependence of eMCE in MnSi^24^, providing a useful set of information for theoretical studies. MnSi is a chiral magnet with a helical magnetic order in the zero field^26,27^. This material and its sister compounds are known for the magnetic-skyrmion crystal phase^28–30^. A recent experiment finds that MnSi also shows nonreciprocal response similar to the eMCE but with a non-monotonic magnetic field dependence^24^; similar behavior also appears in CrNb_3_S_6_^25^. These papers report a non-monotonic temperature dependence of the eMCE, which shows a maximum at around the magnetic transition temperature. The result implies the importance of magnetic fluctuation. However, the microscopic mechanism on how the magnetic fluctuation produces nonreciprocity remains elusive.

In this work, we theoretically study the nonreciprocal transport phenomena of electrons focusing on an asymmetric scattering by local magnetic correlation. We find the magnetic correlation in chiral magnets causes asymmetric scattering in the leading order of the scattering. The asymmetry produces a nonreciprocal response of electric current in nonmagnetic systems^31^. In contrast to Isobe et al.^31^, the magnetic scattering produces a larger nonreciprocal current because the asymmetric scattering appears in the leading order. Using the semiclassical Boltzmann theory, we show the magnitude of the nonreciprocal current is consistent with that in the experiment. Moreover, the temperature and magnetic field dependences reproduce the experiment. The consistency between the experiment and our theoretical results provides strong evidence of the extrinsic mechanism for nonreciprocal electric current.

## Results

### Asymmetric scattering rate

To study how the electron scattering produce nonreciprocal response, we here consider a model with itinerant electrons and localized spins coupled by exchange interaction. The Hamiltonian is

2 $$H=\sum _{{\bf{k}}\sigma }{\varepsilon }_{{\bf{k}}\sigma }{c}_{{\bf{k}}\sigma }^{\dagger }{c}_{{\bf{k}}\sigma }+\frac{J}{N}\sum _{\substack{i,{\bf{k}},\\ \alpha ,\beta }}{\gamma }_{i{\bf{k}}}{{\bf{S}}}_{i}\cdot {c}_{{\bf{k}}\alpha }^{\dagger }{{\boldsymbol{\sigma }}}_{\alpha \beta }{c}_{{\bf{k}}\beta },$$

where *c*_**k***σ*_ (${c}_{{\bf{k}}\sigma }^{\dagger }$) are respectively the annihilation (creation) operator of itinerant and localized electrons, ***σ*** ≡ (*σ*^*x*^, *σ*^*y*^, *σ*^*z*^) is the vector of Pauli matrices *σ*^*a*^ (*a* = *x*, *y*, *z*), *ε*_**k***σ*_ = *k*^2^/(2*m*) − *σM* − *μ* is the eigenenergy of itinerant electrons with momentum **k** and spin *σ* = ±1 (+1 for up spin and  −1 for down spin), *k* ≡ ∣**k**∣, ${\gamma }_{i{\bf{k}}}\equiv {e}^{{\rm{i}}{\bf{k}}\cdot {{\bf{r}}}_{i}}$, *J* is the Kondo coupling between the localized spins and the itinerant electrons, and **S**_*i*_ is the localized moment at **r**_*i*_. Here, we assumed the magnetization is along the *z* axis. This model is a classical- spin Kondo lattice model if the localized spins exists on every site, and is a Kondo impurity model if the spins exist only on a few sites *N*_*s*_ ≪ *N*.

We calculate the scattering rate of electrons by the localized spins using Born approximation. In the first Born approximation, the scattering rate ${W}_{{\bf{k}}\sigma ,{\bf{k}}^{\prime} \sigma ^{\prime} }$ of electrons from the **k***σ* state to the ${\bf{k}}^{\prime} \sigma ^{\prime}$ state reads:

3 $${W}_{{\bf{k}}\sigma ,{\bf{k}}^{\prime} \sigma ^{\prime} }=\frac{2\pi {J}^{2}}{{N}^{2}}\sum _{\substack{i,j,\\ a,b}}{S}_{i}^{a}{S}_{j}^{b}{\sigma }_{\sigma \sigma ^{\prime} }^{a}{\sigma }_{\sigma ^{\prime} \sigma }^{b}{e}^{{\rm{i}}({\bf{k}}^{\prime} -{\bf{k}})\cdot ({{\bf{r}}}_{i}-{{\bf{r}}}_{j})}\delta ({\varepsilon }_{{\bf{k}}\sigma }-{\varepsilon }_{{\bf{k}}^{\prime} \sigma ^{\prime} }).$$

Here we assume that the spins are classical and static, which is justified when the temperature is much higher than the typical energy of exchange interaction. The experimental situation in MnSi discussed below satisfies this condition. A recent work point outs that the asymmetry in the scattering rate ${W}_{{\bf{k}},{\bf{k}}^{\prime} }\,\ne \,{W}_{-{\bf{k}},-{\bf{k}}^{\prime} }$ produces the nonreciprocity in the electron transport^31^ (Isobe et al.^31^ considered spinless fermions.). Therefore, we focus on a similar asymmetry in ${W}_{{\bf{k}}\sigma ,{\bf{k}}^{\prime} \sigma ^{\prime} }$. The asymmetric part of the scattering rate (${W}_{{\bf{k}}\sigma ,{\bf{k}}^{\prime} \sigma ^{\prime} }^{-}\equiv =({W}_{{\bf{k}}\sigma ,{\bf{k}}^{\prime} \sigma }-{W}_{-{\bf{k}}\sigma ,-{\bf{k}}^{\prime} \sigma })/2$) reads

4 $${W}_{{\bf{k}}\sigma ,{\bf{k}}^{\prime} \sigma ^{\prime} }^{-}=\frac{2\pi {J}^{2}}{{N}^{2}}\sigma {\delta }_{\sigma ,\bar{\sigma }^{\prime} }\mathop{\sum }\limits_{i,j}^{{N}_{s}}\sin (({\bf{k}}-{\bf{k}}^{\prime} )\cdot {{\bf{r}}}_{ij}){({{\bf{S}}}_{i}\times {{\bf{S}}}_{j})}_{z}\delta ({\varepsilon }_{{\bf{k}}\sigma }-{\varepsilon }_{{\bf{k}}^{\prime} \sigma ^{\prime} }).$$

Here, **r**_*ij*_ = **r**_*i*_ − **r**_*j*_ and $\bar{\sigma }=-\sigma$; we assumed *ε*_**k***σ*_ = *ε*_−**k***σ*_. This asymmetric scattering vanishes when *N*_*s*_ = 1; the sine function is always zero because **S**_1_ × **S**_1_ = **0**. Therefore, multiple spin scattering is necessary for the non-zero asymmetric scattering.

In the two spin case, the scattering rate reads

5 $${W}_{{\bf{k}}\sigma ,{\bf{k}}^{\prime} \sigma ^{\prime} }^{-}=\frac{4\pi {J}^{2}}{{N}^{2}}\sigma {\delta }_{\sigma ,\bar{\sigma }^{\prime} }\sin \left(({\bf{k}}-{\bf{k}}^{\prime} )\cdot {{\bf{r}}}_{12}\right){\left({{\bf{S}}}_{1}\times {{\bf{S}}}_{2}\right)}_{z}\delta ({\varepsilon }_{{\bf{k}}\sigma }-{\varepsilon }_{{\bf{k}}^{\prime} \sigma ^{\prime} }).$$

**Fig. 1:**
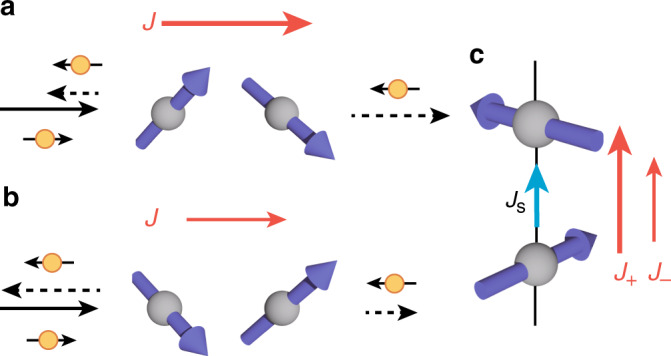
Nonreciprocal transport by magnetic scattering. **a**, **b** Schematic figure of magnetic scattering by a two-spin cluster with finite vector spin chirality. The backward scattering by the two-spin spin cluster scatters incoming electron with up spin (the electron at the light bottom of the figure) depends on the vector spin chirality; less electrons are scattered backward in **a** compared to **b**. The weaker backward scattering in **a** results in a larger current compared to **b**. **c** Nonreciprocal spin current for *S*^*z*^ in the paramagnetic case. The spin current of electrons *J*_s_ flow along the direction of the supercurrent of magnetic moments because of the difference between the current for up-spin electrons and down-spin ones.

Hence, the asymmetry appears when a non-zero vector spin chirality exists, i.e., when the two spins are non-collinear. A previous work on multiferroics in insulators point outs the relation of the local spin current  ∝ **S**_*i*_ × **S**_*j*_ and electric polarization^6^. From a similar viewpoint, our result shows the local spin current scatters electrons asymmetrically depending on the spins (Fig. 1c). In addition, the result implies a finite magnetization is necessary for the nonreciprocity because the asymmetric scattering rate in Eq. (5) has the opposite signs for ${W}_{{\bf{k}}\uparrow ,{\bf{k}}^{\prime} \downarrow }^{-}$ and ${W}_{{\bf{k}}\downarrow ,{\bf{k}}^{\prime} \uparrow }^{-}$. Therefore, the asymmetry cancels when the itinerant electrons are paramagnetic (*M* = 0). In short, the above result implies nonreciprocity in the conductivity appears in a magnet when both the vector spin chirality and magetization are nonzero.

We note that ${W}_{{\bf{k}}\sigma ,{\bf{k}}^{\prime} \sigma ^{\prime} }^{-}$ appears in the first Born approximation. This feature is in contrast to Isobe et al.^31^ where ${W}_{{\bf{k}}\sigma ,{\bf{k}}^{\prime} \sigma ^{\prime} }^{-}$ appears from the second Born terms, i.e., higher-order in the perturbation. For the non-magnetic scatterers in time-reversal symmetric system, ${W}_{{\bf{k}}\sigma ,{\bf{k}}^{\prime} \sigma ^{\prime} }^{-}$ is related to the skew scattering ${W}_{{\bf{k}},{\bf{k}}^{\prime} }^{{\rm{s}}}=({W}_{{\bf{k}},{\bf{k}}^{\prime} }-{W}_{{\bf{k}}^{\prime} ,{\bf{k}}})/2$ by ${\mathcal{T}}$^31^. The skew scattering is prohibited in the first Born approximation because of the Hermiticity of the impurity potential. Therefore, the second-order term is the leading order. In contrast, the magnetic scattering considered here breaks ${\mathcal{T}}$. This difference of the symmetry allows non-zero ${W}_{{\bf{k}}\sigma ,{\bf{k}}^{\prime} \sigma ^{\prime} }^{-}$ in the leading-order Born approximation. This result also implies that the magnetic scattering produces a larger nonreciprocal response.

In MnSi, the spin–orbit interaction modifies the electronic bands, for instance, by a term such as *H*_SOI_ = *λ***k** ⋅ ***σ***. These terms, however, merely modifies our result by adding correction to the results above (see Supplementary Note 2). Therefore, we neglect the spin-orbit interaction on the itinerant electrons.

### Boltzmann theory for nonreciprocal currents

To study how ${W}_{{\bf{k}}\sigma ,{\bf{k}}^{\prime} \sigma ^{\prime} }^{-}$ contributes to the eMCE, we calculate the conductivity using the semiclassical Boltzmann theory. Using the relaxation time approximation, the Boltzmann equation reads

6 $${{e}}{\bf{E}}\cdot {{\boldsymbol{\nabla }}}_{{\bf{k}}}{f}_{{\bf{k}}\sigma }=-\frac{{f}_{{\bf{k}}\sigma }-{f}_{{\bf{k}}\sigma }^{0}}{\tau }+\sum _{{\bf{k}}^{\prime} ,\sigma ^{\prime} }{W}_{{\bf{k}}\sigma ,{\bf{k}}^{\prime} \sigma ^{\prime} }^{-}({f}_{{\bf{k}}^{\prime} \sigma ^{\prime} }-{f}_{{\bf{k}}\sigma }).$$

Here, *e* < 0 is the elementary charge, **E** = (*E*_*x*_, *E*_*y*_, *E*_*z*_) is the applied electric field, and *f*_**k***σ*_ is the electron density for the electrons with momentum **k** and spin *σ*. The relaxation time includes the contribution from non-magnetic scattering process as well as the symmetric scattering ${W}_{{\bf{k}}\sigma ,{\bf{k}}^{\prime} \sigma ^{\prime} }^{+}\equiv ({W}_{{\bf{k}}\sigma ,{\bf{k}}^{\prime} \sigma ^{\prime} }+{W}_{-{\bf{k}}\sigma ,-{\bf{k}}^{\prime} \sigma ^{\prime} })/2$ by the magnetic moments. For simplicity, we focus on the case **E** = (0, 0, *E*). In addition, we assume

7 $${W}_{{\bf{k}}\sigma ,{\bf{k}}^{\prime} \sigma ^{\prime} }^{-}=\left\{\begin{array}{ll} 0&({\rm{if}}\ \sigma\,=\,\sigma ^{\prime} )\\ 2\pi \sigma c({k}_{z}-{k}_{z}^{\prime})\delta ({\varepsilon }_{{\bf{k}}\sigma }-{\varepsilon }_{{\bf{k}}^{\prime} \sigma ^{\prime} })&({\rm{if}}\ \sigma \,\ne\, \sigma ^{\prime} )\end{array}\right.,$$

where $c=\frac{{J}^{2}}{N}{\chi }_{{\rm{v}}}$ is a real constant and ${\chi }_{{\rm{v}}}\equiv \langle {({{\bf{S}}}_{i}\times {{\bf{S}}}_{j})}_{z}\rangle$ is the thermal average of the *z* component of the vector spin chirality between the nearest-neighbor spins along the *z* axis. This asymmetric scattering term corresponds to the thermal average of the *k* ≪ 1 case of the two-spin impurity cluster in Eq. (5).

We solve the Boltzmann equation in Eq. (6) with the scattering rate in Eq. (7) by expanding *f*_**k***σ*_ up to the second order in **E** and linear order in *c*^31^. Within this approximation, the nonreciprocal current reads

8 $${J}_{z}^{(2)}=-\frac{144\pi }{5}\frac{\tau m}{e{\mu }^{2}}cM{\sigma }_{0}^{2}{E}^{2},$$

where $2{\sigma }_{0}=\frac{4{{\rm{e}}}^{2}\tau \mu }{3mn}$ is the linear conductivity of electrons at *M* = 0 and *c* = 0. Here, *n* is the density of state at the Fermi level and we assumed *μ* ≫ *M*. Hence, the scattering by the two spins produce non-reciprocal current proportional to *c* and magnetic polarization of the itinerant electrons *M*.

We also note that the two spin scattering produces the spin current. Using the same formalism, we find the spin current for *S*^*z*^ reads

9 $${J}_{z}^{z}=-\frac{54\pi \hslash }{5}\frac{\tau m}{{{\rm{e}}}^{2}\mu }c\ {\sigma }_{0}^{2}{E}^{2}.$$

Unlike the charge current, the spin current appears without the spin polarization. Therefore, a paramagnet with the chiral spin correlation produces a finite spin current by simply flowing electric current. This spin current is qualitatively different from the spin current induced by the interfacial effect^32^, where the sign and magnitude of the spin current depends on the interface between the helical order and the leads. In contrast, the spin current in Eq. (9) is a phenomenon occuring in the bulk material.

### Nonreciprocal charge current in chiral magnets

In the above mechanism, the nonreciprocal current depends on temperature and magnetic field via that of the magnetization and vector spin chirality. To investigate the dependence of nonlinear conductance, we here consider a classical ferromagnetic Heisenberg model on a cubic lattice with Dzyaloshinskii–Moriya interaction^27,30,33^,

10 $${H}_{{\rm{cm}}}=-J\sum _{\langle i,j\rangle }{{\bf{S}}}_{i}\cdot {{\bf{S}}}_{j}-\frac{D}{2}\sum _{\langle i,j\rangle }{{\bf{r}}}_{ji}\cdot {{\bf{S}}}_{i}\times {{\bf{S}}}_{j}-h\sum _{i}{S}_{i}^{z}.$$

**Fig. 2:**
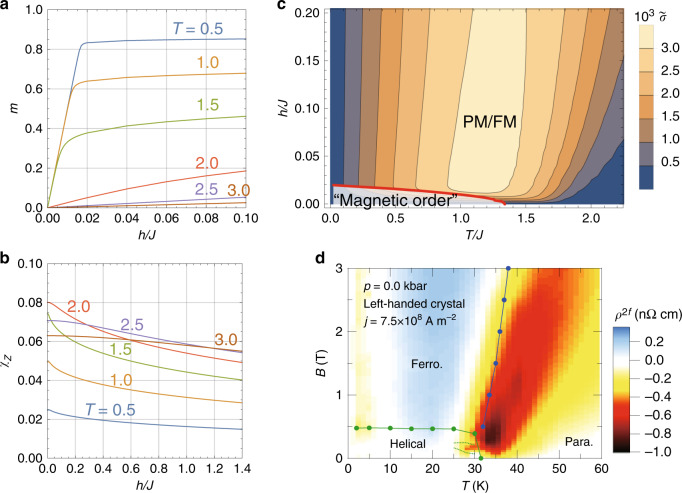
Magnetic and transport properties of a three-dimensional chiral magnet. The magnetic-field dependence of **a** magnetization *M* and **b** vector spin chirality *χ*_*z*_ for different temperature *T*. **c** is the contour plot of $\tilde{\sigma }=M{\chi }_{z}$. The results are for *D*/*J* = 0.2. The red line is the phase boundary between the ordered and paramagnetic (PM/FM) phases, which is determined by *λ* + *D*^2^/(4*J*) > −10^−4^. See Method section for details. **d** The contour plot of second harmonic resistivity *ρ*^2*f*^( ∝ *σ*^(2)^). Reproduced from ref. ^24^.

Here, the sum is over the nearest-neighbor bonds. Figure 2 shows the magnetic and transport properties of the above model; all results are obtained using Onsager’s reaction field theory (See Methods section). Figure 2a is the plot of the magnetization to the magnetic field. The result shows a ferromagnetic magnetization curve below *T* ≲ 2*J*, which decreases monotonically with increasing temperature. In contrast, the vector spin chirality *χ*_*z*_ shows a non-monotonic temperature dependence. Figure 2b shows the magnetic field dependence of ∣*χ*_*z*_∣ for different *T*. When *T*/*J* ≲ 1, the field-induced magnetization suppresses ∣*χ*_*z*_∣ → 0 as *T* → 0. With increasing temperature, the thermal fluctuation increase ∣*χ*_*z*_∣ by suppressing the magnetization. The maximum is around *T*/*J* ~ 2−3 depending on *h*; the maximum tends to move to a higher *T* as *h* increases. Further increase of the temperature reduces the spin chirality because the thermal fluctuation dominates over the exchange interactions between the spins.

Equation (8) shows the nonreciprocal conductivity *σ*^(2)^ is proportional to ${\tilde{\sigma }}^{(2)}\equiv M{\chi }_{z}$. Figure 2c shows the contour plot of ${\tilde{\sigma }}^{(2)}$ in the *T* − *h* plane. In the low temperature region, the result shows a small ${\tilde{\sigma }}^{(2)}$ owing to the suppression of the vector chirality. With increasing the temperature, ${\tilde{\sigma }}^{(2)}$ increases due to the increase of *χ*_*z*_ with a maximum around *T*/*J* ~ 1.5−2; ${\tilde{\sigma }}^{(2)}$ then decreases because both *M* and *χ*_*z*_ is suppressed by the thermal fluctuation when *T*/*J* ≫ 1. Figure 2c also shows the increase of the maximum with increasing the magnetic field. This is related to the increase of the maximum of *χ*_*z*_ discussed above. We find these behaviors remain robust even when we take into account the temperature dependence of the relaxation time *τ* (see Supplementary Note 1). They are also consistent with the experiment in MnSi as shown in Fig. 2d^24^.

## Discussion

To summarize, we studied the nonreciprocity of electric current produced by spin-cluster scattering. We find that the scattering process involving two spins cause an asymmetric scattering, which is proportional to the vector spin chirality. This effect appears at the leading order in the impurity scattering, i.e., within the first Born approximation. Therefore, we expect a large asymmetry in the scattering rate. Using the semiclassical Boltzmann theory, we find that this asymmetry produces nonreciprocal transport of electrons; *σ*^(2)^ is proportional to the vector spin chirality and spin polarization of itinerant electrons. We also find the chiral-spin scattering produces nonreciprocal spin current, in contrast to recently-reported boundary effect^32^. As a consequence, *σ*^(2)^ shows a non-monotonic temperature with a maximum around *T*/*J* ~ 1. This trend is consistent with the recent experiments in MnSi^24^ and CrNb_3_S_6_^25^. In particular, the overall behavior of *σ*^(2)^ well accounts for the eMCE in MnSi^24^.

The magnitude of the eMCE by the magnetic scattering is also consistent with the experiment in MnSi^24^. A recent experiment of MnSi finds the ratio of linear and nonreciprocal resistivities *γ*(*B*)*IB* ~ 10^−4^ − 10^−5^ with *I* = 10^9^ Am^−2^. When *γ*(*B*)*IB* ≪ 1, the ratio reads *γIB* ~ −*σ*^(2)^*I*/*σ*^2^. We estimate *σ*^(2)^ using Eq. (8) assuming *J* = 10 meV, *D* = 1 meV, *a*_0_ = 4 Å, *m* = 9.109 × 10^−31^ kg, $\rho =1/(2{\mu }_{{\rm{F}}}{a}_{0}^{3}) \sim 1{0}^{39}$ J^−1^ cm^−3^, and *τ* = 10^−13^ s. We use *μ*_F_ = 0.5 eV and *M*(=*gμ*_B_*H*) = 100 meV because the bandwidth is  ~1 eV^34^ and the spin polarization is in the order of 10%^35^. Using these values, we find *σ*^(2)^*I*/*σ*^2^ ~ 2 × 10^−5^. Therefore, the result is roughly comparable with that observed in MnSi.

The quantum effects of the spin such as Kondo effect on the spin cluster scattering are an interesting problem. Within the second Born approximation, the log singularity due to the second-Born approximation is absorbed in the renormalized exchange coupling *J*. Therefore, both the usual symmetric scattering and anti-symmetric scattering are enhanced (reduced) towards the Kondo temperature *T*_K_ for the antiferromagnetic (ferromagnetic) *J*. As the temperature is further lowered, the physics of multi-spin Kondo effect will show up and the nonreciprocal nature there is an important and interesting problem left for future studies.

## Methods

### Boltzmann theory

We used the semiclassical Boltzmann theory to calculate the nonreciprocal current. Assuming the steady state, the semiclassical Boltzmann equation reads

11 $${{e}}{\bf{E}}\cdot {{\boldsymbol{\nabla }}}_{{\bf{k}}}{f}_{{\bf{k}}\sigma }=\sum _{{\bf{k}}^{\prime} ,\sigma ^{\prime} }\left({W}_{{\bf{k}}\sigma ,{\bf{k}}^{\prime} \sigma ^{\prime} }{f}_{{\bf{k}}^{\prime} \sigma ^{\prime} }-{W}_{{\bf{k}}^{\prime} \sigma ^{\prime} ,{\bf{k}}\sigma }{f}_{{\bf{k}}\sigma }\right).$$

Here, *e* < 0 is the elementary charge, **E** = (*E*_*x*_, *E*_*y*_, *E*_*z*_) is the applied electric field, and *f*_**k***σ*_ is the electron density for the electrons with momentum **k** and spin *σ*. For simplicity, we focus on the case **E** = (0, 0, *E*). The first (second) term in the right-hand side of the equation represents the scattering of electrons from ${\bf{k}}^{\prime} \sigma ^{\prime}$ (**k***σ*) to **k***σ* (${\bf{k}}^{\prime} \sigma ^{\prime}$). We approximate the symmetric part of the scattering rate by a relaxation time *τ*. A similar approximation were used elsewhere to study transport phenomena related to a specific scattering term^10,31,36,37^. Within this approximation, the Boltzmann equation reads

12 $${{e}}{\bf{E}}\cdot {{\boldsymbol{\nabla }}}_{{\bf{k}}}{f}_{{\bf{k}}\sigma }=-\frac{{f}_{{\bf{k}}\sigma }-{f}_{{\bf{k}}\sigma }^{0}}{\tau }+\sum _{{\bf{k}}^{\prime} ,\sigma ^{\prime} }{W}_{{\bf{k}}\sigma ,{\bf{k}}^{\prime} \sigma ^{\prime} }^{-}({f}_{{\bf{k}}^{\prime} \sigma ^{\prime} }-{f}_{{\bf{k}}\sigma }).$$

Here, we assume the form of asymmetric scattering rate to be

13 $${W}_{{\bf{k}}\sigma ,{\bf{k}}^{\prime} \sigma ^{\prime} }^{-}=\left\{\begin{array}{ll}0&({\rm{if}}\ \sigma\, =\,\sigma ^{\prime} )\\ 2\pi \sigma c({k}_{z}-{k}_{z}^{\prime})\delta ({\varepsilon }_{{\bf{k}}\sigma }-{\varepsilon }_{{\bf{k}}^{\prime} \sigma ^{\prime} })&({\rm{if}}\ \sigma \,\ne\, \sigma ^{\prime} )\end{array}\right.$$

where $c=\frac{{J}^{2}}{N}{\chi }_{{\rm{v}}}$ is a real constant and ${\chi }_{{\rm{v}}}\equiv \langle {({{\bf{S}}}_{i}\times {{\bf{S}}}_{j})}_{z}\rangle$ is the thermal average of the *z* component of the vector spin chirality between the nearest-neighbor spins along the *z* axis.

This asymmetric scattering term corresponds to the *k* ≪ 1 case of the two-spin impurity cluster in Eq. (3). It also applies to the paramagnetic phase of the Kondo lattice models where the correlation length between the localized moments are short. In this case, the magnetic moments in Eq. (3) should be replaced by the thermal average,

14 $${W}_{{\bf{k}}\sigma ,{\bf{k}}^{\prime} \bar{\sigma }}^{-}=\frac{2\pi {J}^{2}\sigma }{{N}^{2}}\sum _{i,j}({\bf{k}}-{\bf{k}}^{\prime} )\cdot {{\bf{r}}}_{ij}\langle {({{\bf{S}}}_{i}\times {{\bf{S}}}_{j})}_{z}\rangle \delta ({\varepsilon }_{{\bf{k}}\sigma }-{\varepsilon }_{{\bf{k}}^{\prime} \sigma ^{\prime} }).$$

Here, the sum is over all localized moments in the system. This sum is reduced to the sum over nearest-neighbor bonds when the correlation length is similar or less than the lattice spacing, i.e., $\langle {({{\bf{S}}}_{i}\times {{\bf{S}}}_{j})}_{z}\rangle \sim 0$ for further-neighbor bonds. Assuming $\langle {({{\bf{S}}}_{i}\times {{\bf{S}}}_{j})}_{z}\rangle ={\chi }_{z}\ne\, 0$ only for the nearest-neighbor bonds along the *z* axis, the constant *c* reads *c* = *J*^2^*χ*_*z*_ (we chose the unit of length as ∣**r**_*ij*_∣ = 1 for the nearest-neighbor bonds).

We solve the Boltzmann equation in Eq. (6) with the scattering rate in Eq. (7) by expanding *f*_**k***σ*_ up to the second order in **E** and linear order in *c*^31^; ${f}_{{\bf{k}}\sigma }={f}_{{\bf{k}}\sigma }^{0}+{\sum }_{i = 1,2,j = 0,1}{g}_{{\bf{k}}\sigma }^{(i,j)}$ where ${f}_{{\bf{k}}\sigma }^{0}=1/(1+{e}^{\beta {\varepsilon }_{{\bf{k}}\sigma }})$ is the Fermi distribution function and ${g}_{{\bf{k}}\sigma }^{(i,j)}$ is the deviation from the equilibrium distribution in the *i*th-order in *E* and *j*th order in *c*. We find

15 $${g}_{{\bf{k}}\sigma }^{(1,0)}=-\tau {{e}}{\bf{E}}\cdot {{\boldsymbol{\nabla }}}_{k}{f}_{{\bf{k}}\sigma }^{0}=\tau {{e}}{\bf{E}}\cdot {{\bf{v}}}_{{\bf{k}}\sigma }\delta ({\varepsilon }_{{\bf{k}}\sigma }),$$

16 $${g}_{{\bf{k}}\sigma }^{(1,1)}=\tau \int\frac{d{\bf{k}}^{\prime} }{{(2\pi )}^{3}}{W}_{{\bf{k}}\sigma ,{\bf{k}}^{\prime} \sigma ^{\prime} }^{-}\left({g}_{{\bf{k}}^{\prime} \sigma ^{\prime} }^{(1,0)}-{g}_{{\bf{k}}\sigma }^{(1,0)}\right),$$

17 $${g}_{{\bf{k}}\sigma }^{(2,0)}=-\tau {{e}}{\bf{E}}\cdot {{\boldsymbol{\nabla }}}_{{\bf{k}}}{g}_{{\bf{k}}\sigma }^{(1,0)},$$

18 $${g}_{{\bf{k}}\sigma }^{(2,1)}=-\tau {{e}}{\bf{E}}\cdot {{\boldsymbol{\nabla }}}_{{\bf{k}}}{g}_{{\bf{k}}\sigma }^{(1,1)}+\tau \int\frac{d{\bf{k}}^{\prime} }{{(2\pi )}^{3}}{W}_{{\bf{k}}\sigma ,{\bf{k}}^{\prime} \sigma ^{\prime} }^{-}\left({g}_{{\bf{k}}^{\prime} \sigma ^{\prime} }^{(2,0)}-{g}_{{\bf{k}}\sigma }^{(2,0)}\right).$$

In the Boltzmann theory, the current along the *z* axis reads

19 $${J}_{z}={\rm{e}}\sum _{\sigma }\int\frac{d{\bf{k}}}{{(2\pi )}^{3}}{v}_{{\bf{k}}\sigma }^{z}{f}_{{\bf{k}}\sigma }={\rm{e}}\sum _{\sigma }\sum _{i,j}\int\frac{d{\bf{k}}}{{(2\pi )}^{3}}{v}_{{\bf{k}}\sigma }^{z}{g}_{{\bf{k}}\sigma }^{(i,j)}.$$

Here, ${\rho }_{\sigma }\equiv \int\frac{d{\bf{k}}}{{(2\pi )}^{3}}\delta ({\varepsilon }_{{\bf{k}}\sigma })$ is the density of states for the electrons with spin *σ*. Therefore, the nonreciprocal current in ${\mathcal{O}}({E}^{2})$ reads

20 $${J}_{z}^{(2)}={\rm{e}}\sum _{\sigma }\int\frac{d{\bf{k}}}{{(2\pi )}^{3}}{v}_{{\bf{k}}\sigma }^{z}\left[{g}_{{\bf{k}}\sigma }^{(2,1)}+{g}_{{\bf{k}}\sigma }^{(2,2)}\right].$$

The ${g}_{{\bf{k}}\sigma }^{(2,1)}$ term contributes to the nonreciprocal current when the electronic band is asymmetric due to the absence of both time and spatial inversion symmetries^20^; this term vanishes in our case. Therefore, we here focus on the second term related to ${g}_{{\bf{k}}\sigma }^{(2,2)}$. The nonreciprocal current reads

21 $${J}_{z}^{(2)}=-\frac{16\pi }{5}m\tau {\rm{e}}{\rho }_{+}{\rho }_{-}cM\frac{4{\mu }^{2}-3{M}^{2}}{{\mu }^{2}-{M}^{2}}{\left(\frac{\tau {\rm{e}}E}{m}\right)}^{2},$$

22 $$\quad \sim -\frac{144\pi }{5}\frac{\tau m}{{\rm{e}}{\mu }^{2}}cM{\sigma }_{0}^{2}{E}^{2}.$$

Here, ${\rho }_{\sigma }=\frac{m}{2{\pi }^{2}}\sqrt{2m(\mu +\sigma M)}$ is the density of states for the electrons with spin *σ*. In the second line, we assumed *μ* ≫ *M* and expanded up to the leading order in *M*; $2{\sigma }_{0}=\frac{4{e}^{2}\tau \mu }{3m}$ is the linear conductivity of electrons at *M* = 0 and *c* = 0. Hence, the nonreciprocal current is proportional to the vector spin chirality *c* = *J*^2^*χ*_*z*_ and magnetic polarization of the itinerant electrons *M*.

Similarly, the spin current reads

23 $${J}_{z}^{(2)}=\frac{\hslash }{2}\sum _{\sigma }\int\frac{d{\bf{k}}}{{(2\pi )}^{3}}\sigma {v}_{{\bf{k}}\sigma }^{z}\left[{g}_{{\bf{k}}\sigma }^{(2,1)}+{g}_{{\bf{k}}\sigma }^{(2,2)}\right],$$

24 $$\quad =-\frac{4\pi \hslash {\tau }^{3}{{\rm{e}}}^{2}}{m}c{\rho }_{+}{\rho }_{-}\mu \left\{1+\frac{1}{5}\left(\frac{{\mu }^{2}+3{M}^{2}}{{\mu }^{2}-{M}^{2}}\right)\right\},$$

25 $$\quad \sim -\frac{54\pi \hslash }{5}\frac{\tau m}{{{\rm{e}}}^{2}\mu }c\ {\sigma }_{0}^{2}{E}^{2}.$$

The last equation is the result for *μ* ≫ *M*. The last equation implies the chiral correlation produces spin current in a paramagnetic phase without magnetization. A study on electric polarization by spin canting finds the polarization is parallel to **r**_*ij*_ × **j**_s_ where **j**_s_ ∝ **S**_*i*_ × **S**_*j*_ is the supercurrent of spin current^6^. In contrast, our result finds the component of **j**_s_ parallel to **r**_*ij*_ is proportional to the spin current of electrons (Fig. 1a).

### Magnetic phase diagram

Onsager’s reaction field theory is used to calculate the magnetization and the vector spin chirality under external magnetic field^38–40^. This method incorporates the ∑_*i*_∣**S**_*i*_∣^2^ = *N*_s_ constraint by introducing a Lagrange’s multiplier *λ*. The effective Hamiltonian reads

26 $${H}_{{\rm{eff}}}={\tilde{H}}_{{\rm{cm}}}+\lambda \sum _{i}| {{\bf{S}}}_{i}{| }^{2}.$$

Using this method, we find the magnetization and vector spin chirality are given by

27 $${m}_{z}=-\frac{h}{2\lambda },$$

and

28 $${\chi }_{z}=\frac{2DT}{3{\pi }^{2}}\int\nolimits_{0}^{\Lambda }\frac{dq\ {q}^{4}}{{(J{q}^{2}-\lambda )}^{2}-{D}^{2}{q}^{2}}.$$

Here, **q** = (*q*_*x*_, *q*_*y*_, *q*_*z*_) is the wavenumber of the classical spin wave modes, *q* = ∣**q**∣, *λ* is determined by

29 $$1-\frac{{h}^{2}}{4{\lambda }^{2}}=\int\frac{d{\bf{q}}}{{(2\pi )}^{3}}T\ {\rm{Tr}}\left(\frac{1}{\lambda -{J}_{{\bf{q}}}}\right),$$

and

30 $${J}_{{\bf{q}}}=\left(\begin{array}{lll}J{q}^{2}&{\rm{i}}D{q}_{z}&-{\rm{i}}D{q}_{y}\\ -{\rm{i}}D{q}_{z}&J{q}^{2}&{\rm{i}}D{q}_{x}\\ {\rm{i}}D{q}_{y}&-{\rm{i}}D{q}_{x}&J{q}^{2}\end{array}\right).$$

This model does not show a phase transition for arbitrary choices of *h* and *T* when *D* ≠ 0. This is an artifact of the approximation used in *J*_**q**_, where the model has a *SO*(3) rotational symmetry in the momentum space. In the lattice model, however, the small anisotropy due to discrete rotational symmetry breaks the *SO*(3) symmetry. To give an idea on the ordering by the anisotropy, we defined the system is magnetically ordered if *λ*(*T*, *h*) + *D*^2^/(4*J*) < −10^−4^. Here,  −*D*^2^/(4*J*) is the ground state energy. In Fig. 2c, we plot the phase boundary by the red solid line.

## Supplementary information

Supplementary Information

## Data Availability

The authors declare that all data supporting the findings of this study are available within the paper.
