# Supplementary material for: Anomalous electrical magnetochiral effect by chiral spin-cluster scattering
Source: Nat Commun. 2020 Jun 12;11:2986. doi: 10.1038/s41467-020-16751-2 (PMC7293218; doi:10.1038/s41467-020-16751-2)
Supplement: Supplementary file 1 — Supplementary Information [file 41467_2020_16751_MOESM1_ESM.pdf]

Supplementary Information to
"*Anomalous electrical magnetochiral effect by chiral spin-cluster scattering*"

Hiroaki Ishizuka[1] and Naoto Nagaosa[1, 2]

[1]*Department of Applied Physics, The University of Tokyo, Bunkyo, Tokyo, 113-8656, JAPAN*
[2]*RIKEN Center for Emergent Matter Sciences (CEMS), Wako, Saitama, 351-0198, JAPAN*

Supplementary Note 1. Temperature dependence of the resistivity

The resistivity of magnetic metals is usually temperature dependent owing to phonon and magnetic scatterings. This is indeed the case for MnSi [1]. To see how the temperature dependence affects our result, we calculated the contour plot of nonreciprocal conductivity assuming the linear conductivity $\sigma \propto \tau_0/(1+\tau_0 T)$. Figure 1 shows the result for $\tau_0 = 1/4$ and $1/2$. The overall feature of the contour plot remains the same with that of the constant conductivity: the nonreciprocal conductivity increases with increasing T for $T/J \sim 1$ while it decreases with increasing T above $T/J \gtrsim 1$. This result shows that the qualitative feature of the phase diagram does not depend on the temperature dependence of the linear conductivity.

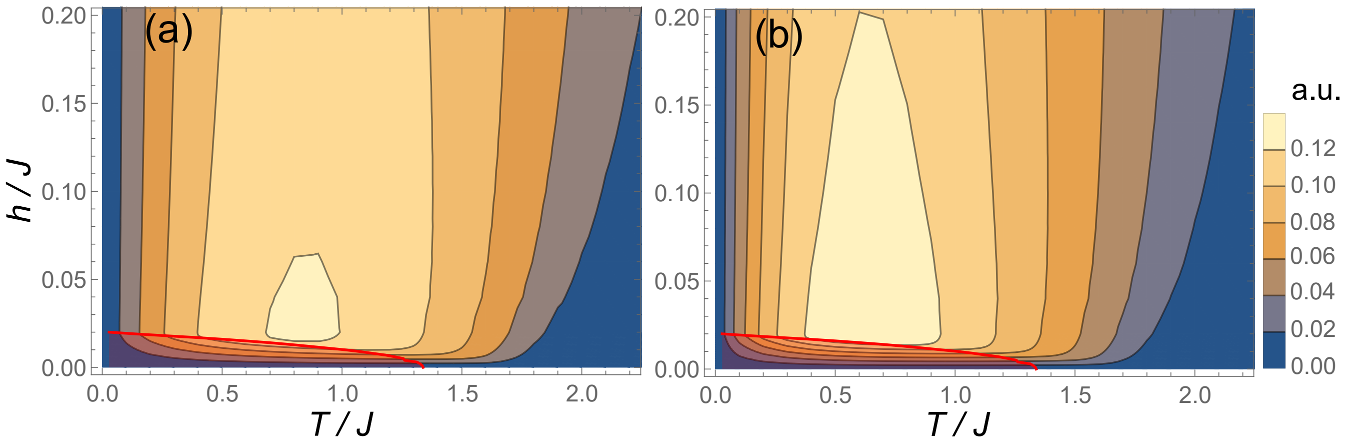

Supplementary Figure 1. Contour plot of nonreciprocal conductivity $\sigma^{(2)}$ for (a) $\tau_0 = 1/4$ and (b) $\tau_0 = 1/2$.

Supplementary Note 2. Effect of spin-orbit interaction on the electronic bands

The spin-orbit interaction often affects the electroic bands, which potentially causes to unconventional properties of the materials. In general, there are two possible contributions from the spin-orbit interaction.

One is the asymmetric deformation of electronic bands. In MnSi, we expect a spin-orbit interaction term $H_{SOI} = \lambda \mathbf{k} \cdot \boldsymbol{\sigma}$. $H_{\rm SOI}$, together with Zeeman shift $H_z = -\mathbf{h} \cdot \boldsymbol{\sigma}$, makes electronic dispersion asymmetric between $\mathbf{k}$ and $-\mathbf{k}$. In this case, the nonreciprocity of resistivity appears even with the usual scattering by impurities and/or spins [2]. This mechanism, however, should be present almost everywhere in the B-T phase diagram in sharp contrast to the experimental observations. Presumably, this is related to the fact that MnSi is a metal; the previous work on band deformation effect in a semiconductor finds a larger nonreciprocity at lower doping [2]. Therefore, the band deformation effect plays a minor role in MnSi.

Another possible origin of nonreciprocity is the asymmetric scattering induced by the asymmetry of eletronic structure. However, in MnSi, the leading order contribution from this effect is likely to be the correction to the mechanism we considered in the main text. To see this, we here focus on the cluster scattering related to ferromagnetic correlation, i.e, $\langle S_i^a S_j^a \rangle$. In the case of $H_{\rm SOI}$ above, the impurity term has to break the π rotation about the two axes perpendicular to the current direction. However, $\langle S_i^a S_j^a \rangle$ is even under the π rotations. Therefore, the ferromagnetic correlation does not contribute to nonreciprocity. Hence, the only possible contribution comes from the cross terms

such as the vector chirality we considered in the main text, i.e., a correction to the mechanism we studied in this paper.

Supplementary References

[1] Yokouchi, T., Kanazawa, N., Kikkawa, A., Morikawa, D., Shibata, K., Arima, T., Taguchi, Y., Kagawa, F., & Tokura, Y. Electrical magnetochiral effect induced by chiral spin fluctuations. Nat. Commun. **8**, 866 (2017).
[2] Ideue, T., Hamamoto, K., Koshikawa, S., Ezawa, M., Shimizu, S., Kaneko, Y., Tokura, Y., Nagaosa, N., & Iwasa, Y. Bulk rectification effect in a polar semiconductor. Nat. Phys. **13**, 578-583 (2017).
